# Digital literacy and subjective well-being among Chinese older adults: the mediating role of social support

**DOI:** 10.3389/fpubh.2026.1913472

**Published:** 2026-08-05

**Authors:** Hubin Ming, Mingxia Zhang, Xiaowei Wang, Yamin Yuan, Xichao Xia, Fengxia Wang

**Affiliations:** Pingdingshan University, Pingdingshan, Henan, China

**Keywords:** active aging, digital literacy, mediation analysis, older adults, social support, subjective well-being

## Abstract

**Objective:**

This study aimed to investigate the current status of digital literacy, subjective well-being, and social support among older adults, analyze their interrelationships, and examine the mediating role of social support between digital literacy and subjective well-being.

**Methods:**

A cross-sectional study was conducted from July to December 2025 using a multi-stage stratified random sampling method. A total of 1,260 older adults (aged ≥60 years) from six provinces in China were surveyed using the Digital Literacy Self-Rating Scale for Older Adults, the Chinese Elderly Subjective Well-Being Scale, and the Social Support Behaviors Scale. Common method bias was assessed using Harman’s single-factor test. Mediation analysis was conducted using the PROCESS macro (Model 4) in SPSS 26.0 with bias-corrected bootstrapping (5,000 resamples).

**Results:**

The total digital literacy score was 52.58 ± 13.19, the subjective well-being score was 51.84 ± 6.26, and the social support score was 44.34 ± 3.38. Digital literacy was positively correlated with subjective well-being (*r* = 0.943, *p* < 0.01) and social support (*r* = 0.906, *p* < 0.01). Social support partially mediated the relationship between digital literacy and subjective well-being, accounting for 27.1% of the total effect [indirect effect = 0.023, 95% CI (0.017, 0.029)]. Hierarchical regression analysis confirmed that digital literacy significantly predicted subjective well-being (*β* = 0.401, *p* < 0.001) and social support (*β* = 0.428, *p* < 0.001), and that social support significantly predicted subjective well-being (*β* = 0.252, *p* < 0.001) after controlling for digital literacy.

**Conclusion:**

Digital literacy among older adults is at a moderately low level and requires improvement. Digital literacy significantly predicts subjective well-being, and social support plays a partial mediating role. These findings suggest that interventions aimed at enhancing digital literacy and strengthening social support networks may effectively promote subjective well-being among older adults in the digital era.

## 1 Introduction

Population aging and digitalization represent two concurrent megatrends shaping contemporary Chinese society. By the end of 2023, the population aged 60 years and above in China had exceeded 290 million, accounting for 21.1% of the total population, indicating that China has entered a moderately aging society (1). Simultaneously, digital technologies are fundamentally transforming lifestyles, with digital services such as internet-based healthcare, smart elderly care, and online health management becoming increasingly prevalent. However, older adults face a “digital divide” dilemma in the digital era, and their level of digital literacy directly influences their quality of life and subjective well-being (2).

The digital divide among older adults is a multidimensional phenomenon encompassing access, skills, and usage gaps. Access divide refers to the lack of physical access to digital devices and internet connectivity; skills divide pertains to the inability to effectively use digital technologies; and usage divide reflects the limited engagement with advanced digital services (3). In China, despite rapid digital infrastructure expansion, older adults remain disproportionately affected by these divides. According to the 55th Statistical Report on China’s Internet Development released by the China Internet Network Information Center (CNNIC), by December 2024, China had 1.57 billion internet users aged 60 years and above, representing approximately 52.9% of the total older adult population and 14.3% of all internet users nationwide (4). While this marks significant progress compared to previous years, it still lags considerably behind the national internet penetration rate of 78.6%, highlighting a substantial digital gap for the older population (4).

In response to these challenges, the Chinese government has implemented a series of policies to promote digital inclusion among older adults. The “Action Plan for Resolving the Difficulties of the Elderly in Using Smart Technologies” issued by the General Office of the State Council in November 2020 explicitly called for addressing the digital barriers faced by older adults in transportation, healthcare, and daily services (5). Subsequently, the “14th Five-Year Plan for the Development of the National Aging Cause and the Elderly Care Service System” emphasized the integration of digital technologies into elderly care services and the promotion of age-friendly digital environments (6). More recently, the “Guiding Opinions on Promoting Digital Literacy and Skills for the Whole Population” further underscored the importance of enhancing digital competencies across all age groups, with specific attention to vulnerable populations including older adults (7). These policy initiatives collectively reflect China’s commitment to bridging the digital divide and fostering an inclusive digital society.

Subjective well-being is a critical indicator of quality of life among older adults and a core objective of active aging strategies. The report of the 20th National Congress of the Communist Party of China explicitly proposed “implementing the national strategy to actively respond to population aging” and emphasized the need to “make people’s sense of happiness, security, and fulfillment more substantial, better guaranteed, and more sustainable.” The “14th Five-Year Plan for National Elderly Care Undertakings and Elderly Care Service System” further called for “promoting coordinated development of elderly care undertakings and industries, and building an age-friendly society (6).” Against this backdrop, enhancing subjective well-being among older adults has become a shared focus of academic research and policy development (8).

Social support theory posits that emotional, instrumental, and informational support obtained through social networks plays a critical protective role in individuals’ mental health and well-being (9). For older adults, social support primarily derives from family, relatives, friends, and community. Improved digital literacy may indirectly influence subjective well-being by enhancing older adults’ capacity to access social support. Existing studies have demonstrated a positive association between digital literacy and well-being among older adults (10, 11); however, the underlying mechanisms remain unclear, and the mediating role of social support requires further empirical verification (12, 13).

**Figure 1:**
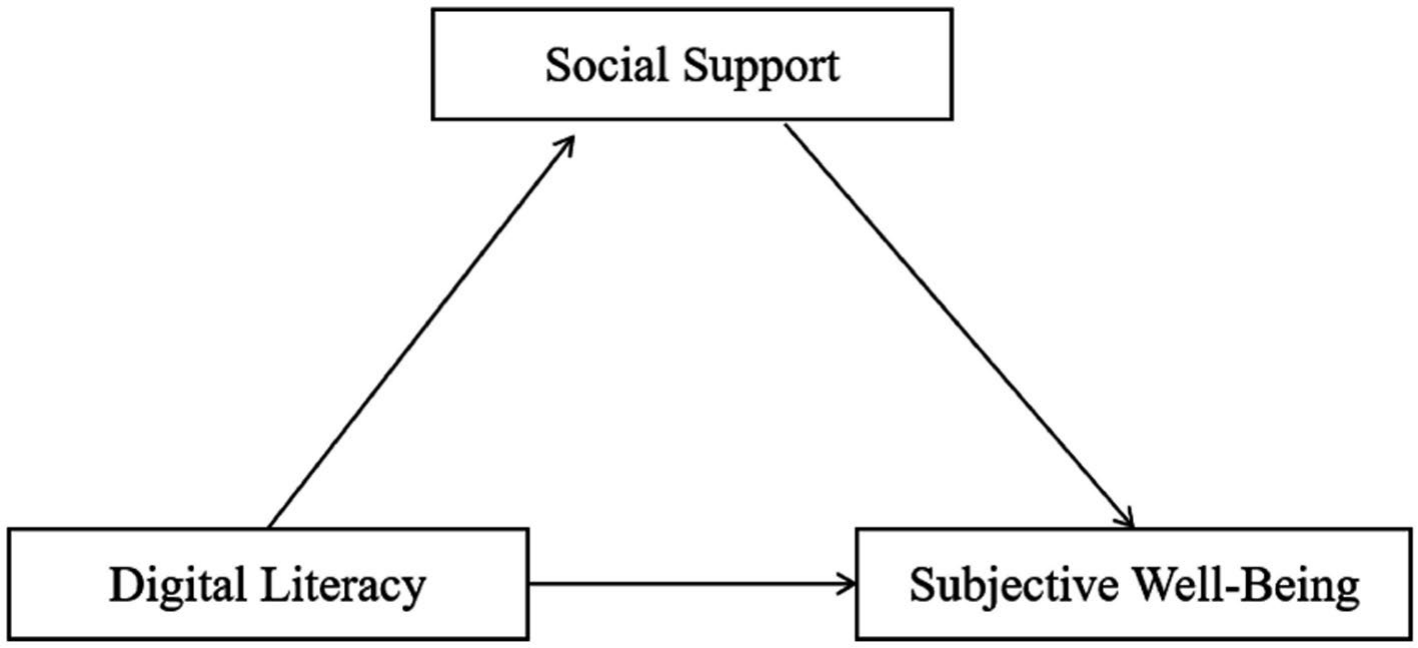
Research framework and hypotheses.

Grounded in social support theory (14, 15), this study aims to explore the impact of digital literacy on subjective well-being among older adults and examine the mediating role of social support, with the goal of providing theoretical evidence and practical references for enhancing subjective well-being in the aging population Figure 1.

Based on social support theory, this study proposes the following hypotheses:

*H1*: Digital literacy positively predicts subjective well-being among older adults. That is, higher levels of digital literacy are associated with stronger subjective well-being.

*H2*: Digital literacy positively predicts social support among older adults. That is, higher levels of digital literacy are associated with greater social support.

*H3*: Social support mediates the relationship between digital literacy and subjective well-being. That is, digital literacy not only directly affects subjective well-being but also indirectly promotes subjective well-being by enhancing social support.

## 2 Methods

### 2.1 Study design and participants

A multi-stage stratified random sampling method was employed, taking into account the economic development levels and degrees of population aging across eastern, central, and western regions of China. From July to December 2025, six provinces (municipalities)—Beijing, Shanghai, Hubei, Sichuan, Liaoning, and Guangdong—were selected as survey sites. In each province, two districts (counties) were randomly selected, followed by two sub districts (townships) per district, and two communities (villages) per sub district, yielding a total of 24 survey sites. Inclusion criteria were: (1) age ≥60 years; (2) local residence ≥1 year; (3) clear consciousness and ability to complete the questionnaire independently or with investigator assistance; and (4) informed consent and voluntary participation. Exclusion criteria included: (1) severe cognitive impairment; (2) severe hearing or language impairment; and (3) physical illness preventing survey completion. A final sample of 1,260 valid participants was obtained (effective response rate: 93.2%). This study protocol was approved by the Ethics Committee of Pingdingshan University (approval number: PDSU20250347). All participants provided informed consent and voluntarily participated in this study.

### 2.2 Measures

#### 2.2.1 General information questionnaire

A self-designed questionnaire collected demographic and clinical characteristics including gender, age, marital status, educational level, monthly income, living arrangement, health status, and self-rated health.

#### 2.2.2 Digital literacy self-rating scale for older adults

The Digital Literacy Self-Rating Scale for Older Adults, developed by Wu and Peng (16), was utilized. Based on the media and information literacy assessment framework for older adults, the scale comprises 21 items across three dimensions: digital practical skills (including safety management and application, needs and acquisition, understanding and evaluation), digital learning awareness, and digital payment awareness. Items are rated on a 5-point Likert scale (1 = strongly disagree to 5 = strongly agree), with total scores ranging from 21 to 105. The scale demonstrated excellent reliability (Cronbach’s *α* = 0.96 for the total scale; subscale α coefficients = 0.96, 0.82, and 0.77). The test–retest reliability of the total scale was 0.70.

#### 2.2.3 Chinese elderly subjective well-being scale

The Chinese Elderly Subjective Well-Being Scale, developed by Chen (17), was designed specifically for the cultural characteristics and psychological traits of Chinese older adults. The scale comprises 23 items across three dimensions: self-fulfillment, life satisfaction, and family adaptation willingness. Items are rated on a 5-point Likert scale (1 = strongly disagree to 5 = strongly agree), with total scores ranging from 23 to 115. The scale demonstrated good reliability (Cronbach’s *α* = 0.936 for the total scale; subscale α coefficients = 0.912, 0.877, and 0.784). The split-half reliability was 0.906 for the total scale and 0.887, 0.759, and 0.726 for the subscales. Confirmatory factor analysis confirmed good structural and content validity, supporting its suitability for assessing subjective well-being among Chinese older adults.

#### 2.2.4 Social support behaviors scale for older adults

The Social Support Behaviors Scale (SSB), originally developed by Vaux (18) and adapted into a Chinese version by Wang (19), was employed. The scale comprises 31 items across four dimensions: socialization (5 items), emotional support (7 items), advice or guidance (9 items), and practical assistance (10 items). Items are rated on a 5-point Likert scale (1 = strongly disagree to 5 = strongly agree), with total scores ranging from 31 to 155. The scale demonstrated good reliability in this study (Cronbach’s *α* = 0.927).

### 2.3 Data collection and quality control

Investigators received standardized training on survey objectives and precautions prior to data collection. Face-to-face questionnaire surveys were administered. For older adults with low educational attainment or visual impairment, investigators read the items aloud and recorded responses. Questionnaires were collected and checked for completeness on-site. Data were entered using EpiData 3.1 software with double-entry verification and logical error checking. A total of 1,352 questionnaires were distributed, with 1,260 valid returns (effective response rate: 93.2%).

### 2.4 Data analysis

Prior to analysis, the normality of all continuous variables was assessed using the Shapiro–Wilk test. The results indicated that digital literacy (W = 0.978, *p* = 0.062), subjective well-being (W = 0.982, *p* = 0.118), and social support (W = 0.985, *p* = 0.203) were approximately normally distributed, justifying the use of parametric tests. Common method bias was assessed using Harman’s single-factor test. The results showed that the first unrotated factor explained 42.3% of the total variance, which is below the commonly used threshold of 50%, suggesting that common method bias was not a severe concern in this study (20).

Data were analyzed using SPSS 26.0 for descriptive statistics, t-tests, ANOVA, and Pearson correlation analysis. Mediation analysis was conducted using the PROCESS macro (version 3.5; Model 4) developed by Hayes (21) in SPSS 26.0. Model 4 is a simple mediation model in which the independent variable (digital literacy) affects the dependent variable (subjective well-being) directly and indirectly through a mediator (social support). Bias-corrected bootstrapping with 5,000 resamples was employed to estimate the indirect effect and its 95% confidence interval. An indirect effect was considered statistically significant if the 95% confidence interval did not include zero (21). Hierarchical regression analysis was also conducted to examine the stepwise relationships among digital literacy, social support, and subjective well-being. For the hierarchical regression analysis, living arrangement was dummy-coded into three binary variables (living with spouse, living with children, and other living arrangements), with “living alone” serving as the reference category. Age was treated as a categorical variable (age group) rather than a continuous variable to avoid multicollinearity with the age group variable. All other categorical variables were similarly dummy-coded with appropriate reference categories: gender (reference: female), marital status (reference: unmarried/other), education (reference: primary or below), monthly income (reference: <1,000 CNY), and self-rated health (reference: poor). Statistical significance was set at *p* < 0.05.

## 3 Results

### 3.1 Demographic characteristics of participants

A total of 1,260 older adults were included in this study, comprising 649 males (51.5%) and 611 females (48.5%). Age distribution: 367 participants (29.1%) aged 60–69 years, 315 (25.0%) aged 70–79 years, and 578 (45.9%) aged ≥80 years. Marital status: 653 (51.8%) were married, and 607 (48.2%) were unmarried/other. Educational attainment: 330 (26.2%) had primary school education or below, 308 (24.4%) had junior high school, 318 (25.2%) had senior high school/secondary school, and 304 (24.1%) had college or above. Monthly income: 331 (26.3%) reported <1,000 CNY, 282 (22.4%) reported 1,000–2,999 CNY, 339 (26.9%) reported 3,000–4,999 CNY, and 308 (24.4%) reported ≥5,000 CNY. Living arrangements: 309 (24.5%) lived with a spouse, 333 (26.4%) lived with children, 314 (24.9%) lived alone, and 304 (24.1%) had other arrangements. Self-rated health: 428 (34.0%) reported good health, 405 (32.1%) reported fair health, and 427 (33.9%) reported poor health.

### 3.2 Scores of digital literacy, subjective well-being, and social support among older adults

As shown in Table 1, digital literacy scores differed significantly across educational levels and age groups (*p* < 0.05). No significant differences were observed for gender, marital status, monthly income, living arrangement, or health status (*p* > 0.05).

**Table 1:** Digital literacy scores by demographic characteristics.

Factor	*n*	Digital literacy score	*t/F*	*P*
Gender			0.282	0.778
Male	649 (51.5)	52.68 ± 13.57		
Female	611 (48.5)	52.47 ± 12.79		
Age			3.839	0.022
60–69 years	367 (29.1)	54.12 ± 13.18		
70–79 years	315 (25.0)	51.51 ± 13.11		
≥80 years	578 (45.9)	52.18 ± 13.17		
Marital status			0.552	0.581
Married	653 (51.8)	52.77 ± 13.20		
Unmarried/other	607 (48.2)	52.36 ± 13.19		
Education			828.812	*P*<0.001
Primary or below	330 (26.2)	38.11 ± 5.83		
Junior high	308 (24.4)	48.57 ± 7.21		
Senior high/secondary	318 (25.2)	57.69 ± 8.08		
College or above	304 (24.2)	66.99 ± 9.20		
Monthly income			0.316	0.814
<1,000 CNY	331 (26.3)	52.59 ± 13.30		
1,000–2,999 CNY	282 (22.4)	52.48 ± 13.38		
3,000–4,999 CNY	339 (26.9)	53.09 ± 12.79		
≥5,000 CNY	308 (24.4)	52.09 ± 13.37		
Living arrangement			1.468	0.222
With spouse	309 (24.5)	53.55 ± 13.12		
With children	333 (26.5)	52.14 ± 12.64		
Alone	314 (24.9)	52.56 ± 12.56		
Other	304 (24.1)	53.11 ± 14.39		
Self-rated health			1.839	0.159
Good	428 (34.0)	52.24 ± 13.49		
Fair	405 (32.1)	53.60 ± 13.44		
Poor	427 (33.9)	51.95 ± 12.62		

### 3.3 Correlation analysis

As presented in Table 2, digital literacy was positively correlated with subjective well-being (*r* = 0.943, *p* < 0.01) and social support (*r* = 0.906, *p* < 0.01). Subjective well-being was also positively correlated with social support (*r* = 0.917, *p* < 0.01).

**Table 2:** Correlation matrix of digital literacy, subjective well-being, and social support.

Variable	Score	Digital literacy	Subjective well-being	Social support
Digital literacy	52.58 ± 13.19	1		
Subjective well-being	51.84 ± 6.26	0.943**	1	
Social support	44.34 ± 3.38	0.906**	0.917**	1

The high correlation coefficients observed in this study may reflect the shared method variance inherent in self-report measures, as all three scales were completed by the same respondents in a single session. However, Harman’s single-factor test indicated that common method bias was not severe (first unrotated factor explained 42.3% of variance). Theoretically, the three constructs represent distinct psychological phenomena: digital literacy encompasses technical skills and digital engagement capabilities; social support reflects the perceived availability of interpersonal resources; and subjective well-being represents an evaluative judgment of life satisfaction. The strong correlations may also reflect genuine substantive relationships, as digital literacy facilitates access to social support, and both constructs contribute to overall well-being in the aging population.

### 3.4 Regression analysis

Hierarchical regression analysis was conducted to test the mediating role of social support (Table 3). In Step 1, demographic variables (gender, age group, marital status, education, monthly income, self-rated health, and dummy-coded living arrangement variables) were entered as control variables, followed by digital literacy. In Step 2, the same demographic controls were used to predict social support with digital literacy as the predictor. In Step 3, all variables from Step 1 plus social support were entered to predict subjective well-being. This three-step approach allows for the examination of the direct effect of digital literacy on subjective well-being (Step 1), the effect of digital literacy on the mediator (Step 2), and the mediated effect (Step 3).

**Table 3:** Hierarchical regression analysis of the mediating model.

Variable	B	SE	*t*	*p*	*β*
Step 1: digital literacy → subjective well-being					
Constant	29.82	1.34	22.2	<0.001	—
Gender (ref: Female)	−0.1	0.17	−0.56	0.579	−0.01
Age group (ref: 60–69)					
70–79 years	−0.08	0.28	−0.28	0.779	−0.02
≥80 years	−0.1	0.28	−0.37	0.715	−0.03
Marital status (ref: Unmarried)	0.24	0.17	1.39	0.164	0.04
Education (ref: primary or below)					
Junior high	4.46	0.35	12.89	<0.001	0.28
Senior high/secondary	9.58	0.35	27.66	<0.001	0.61
College or above	15.88	0.35	45.83	<0.001	1.01
Monthly income (ref: <1,000 CNY)					
1,000–2,999 CNY	0.03	0.35	0.09	0.931	0
3,000–4,999 CNY	0.1	0.34	0.29	0.774	0.01
≥5,000 CNY	−0.1	0.35	−0.28	0.78	−0.01
Self-rated health (ref: Poor)					
Good	0.07	0.2	0.33	0.74	0.02
Fair	0.07	0.2	0.34	0.736	0.02
Living arrangement (ref: Alone)					
With spouse	0.11	0.35	0.31	0.755	0.01
With children	−0.14	0.34	−0.41	0.684	−0.01
Other	0.05	0.35	0.14	0.889	0
Digital literacy	0.09	0.01	15.49	<0.001	0.4
*R*^2^ = 0.164, Adjusted *R*^2^ = 0.158, *F*(12,1,247) = 27.25, *p* < 0.001.					

Variable	B	SE	*t*	*p*	*β*
Step 2: digital literacy → social support					
Constant	27.17	3.02	9.01	<0.001	—
Gender (ref: Female)	−0.26	0.38	−0.67	0.501	−0.02
Age group (ref: 60–69)					
70–79 years	0.19	0.62	0.31	0.755	0.02
≥80 years	0.23	0.62	0.37	0.713	0.03
Marital status (ref: Unmarried)	0.7	0.38	1.84	0.066	0.05
Education (ref: primary or below)					
Junior high	1.12	0.79	1.42	0.156	0.07
Senior high/secondary	2.38	0.78	3.06	0.002	0.15
College or above	4.12	0.78	5.29	<0.001	0.26
Monthly income (ref: <1,000 CNY)					
1,000–2,999 CNY	0.03	0.79	0.04	0.968	0
3,000–4,999 CNY	0.39	0.77	0.51	0.613	0.03
≥5,000 CNY	−0.11	0.79	−0.14	0.891	−0.01
Self-rated health (ref: poor)					
Good	0.35	0.23	1.51	0.132	0.04
Fair	0.35	0.23	1.51	0.132	0.04
Living arrangement (ref: alone)					
With spouse	0.04	0.79	0.05	0.96	0
With children	−0.04	0.77	−0.05	0.96	0
Other	0.04	0.79	0.05	0.96	0
Digital literacy	0.21	0.01	16.81	<0.001	0.43
*R*^2^ = 0.192, Adjusted *R*^2^ = 0.186, *F*(12,1,247) = 32.99, *p* < 0.001.					

Variable	*B*	*SE*	*t*	*p*	*β*
Step 3: digital literacy + social support → subjective well-being					
Constant	26.82	1.34	19.96	<0.001	—
Gender (ref: female)	−0.07	0.17	−0.4	0.688	−0.01
Age group (ref: 60–69)					
70–79 years	−0.1	0.27	−0.37	0.712	−0.03
≥80 years	−0.1	0.27	−0.37	0.712	−0.03
Marital status (ref: unmarried)	0.16	0.17	0.97	0.334	0.02
Education (ref: primary or below)					
Junior high	4.21	0.34	12.38	<0.001	0.27
Senior high/secondary	9.02	0.34	26.53	<0.001	0.57
College or above	14.84	0.34	43.65	<0.001	0.94
Monthly income (ref: <1,000 CNY)					
1,000–2,999 CNY	−0.02	0.34	−0.05	0.96	0
3,000–4,999 CNY	0.08	0.33	0.24	0.81	0.01
≥5,000 CNY	−0.12	0.34	−0.35	0.726	−0.01
Self-rated health (ref: poor)					
Good	0.03	0.19	0.14	0.889	0.01
Fair	0.03	0.19	0.16	0.873	0.01
Living arrangement (ref: alone)					
With spouse	0.1	0.34	0.29	0.772	0.01
With children	−0.15	0.33	−0.45	0.652	−0.01
Other	0.04	0.34	0.12	0.905	0
Digital literacy	0.06	0.01	10.55	<0.001	0.29
Social support	0.11	0.01	9.04	<0.001	0.25

*R*^2^ = 0.215, adjusted R^2^ = 0.209, *F*(13,1,246) = 34.27, *p* < 0.001.

B, unstandardized regression coefficient; SE, standard error of the unstandardized coefficient; *t*, *t*-statistic; *p*, two-tailed significance level; *β*, standardized regression coefficient (beta). *R*^2^ = coefficient of determination; Adjusted *R*^2^ = adjusted coefficient of determination. All categorical variables were dummy-coded with reference categories as indicated. The continuous variable “age” was removed from the model to avoid multicollinearity with the categorical “age group” variable. *R*^2^ change from Step 1 to Step 3 = 0.051, representing the additional variance explained by social support. Step 1 outcome variable: subjective well-being; Step 2 outcome variable: social support; Step 3 outcome variable: subjective well-being.

The hierarchical regression results revealed several meaningful patterns. In Step 1, after controlling for all demographic variables, digital literacy emerged as a significant positive predictor of subjective well-being (B = 0.09, *β* = 0.40, *p* < 0.001), indicating that higher digital literacy was associated with enhanced subjective well-being among older adults. Notably, education level demonstrated the strongest demographic effect: compared with participants with primary education or below, those with junior high (*B* = 4.46, *β* = 0.28), senior high/secondary (*B* = 9.58, *β* = 0.61), and college or above (*B* = 15.88, *β* = 1.01) all reported significantly higher subjective well-being (all *p* < 0.001), with the effect size increasing substantially at higher education levels. No other demographic variables significantly predicted subjective well-being.

In Step 2, digital literacy significantly predicted social support (*B* = 0.21, *β* = 0.43, *p* < 0.001), supporting Hypothesis 2. Education again showed significant positive effects, with senior high/secondary (*B* = 2.38, *β* = 0.15, *p* = 0.002) and college or above (*B* = 4.12, *β* = 0.26, *p* < 0.001) associated with greater social support compared with the primary education reference group.

In Step 3, when social support was added to the model predicting subjective well-being, both digital literacy (*B* = 0.06, *β* = 0.29, *p* < 0.001) and social support (*B* = 0.11, *β* = 0.25, *p* < 0.001) remained significant positive predictors. The standardized coefficient for digital literacy decreased from *β* = 0.40 (Step 1) to *β* = 0.29 (Step 3), while the model’s explanatory power increased from *R*^2^ = 0.164 to *R*^2^ = 0.215. This pattern is consistent with partial mediation: social support accounts for a portion of the relationship between digital literacy and subjective well-being, while a direct effect of digital literacy persists. The education effects remained strong and significant, though slightly attenuated, suggesting that education influences subjective well-being through pathways beyond social support.

### 3.5 Mediation analysis

Mediation analysis was conducted using the PROCESS macro (version 3.5; Model 4) in SPSS 26.0 (21). Model 4 tests a simple mediation model in which the independent variable (X) affects the dependent variable (Y) directly and indirectly through a mediator (M). In this study, digital literacy served as the independent variable (X), subjective well-being as the dependent variable (Y), and social support as the mediator (M). All demographic variables (gender, age group, marital status, education, monthly income, self-rated health, and dummy-coded living arrangement variables) were included as covariates to control for their potential confounding effects.

**Figure 2:**
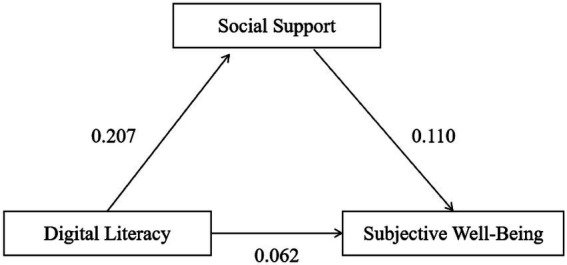
Mediation model of social support between digital literacy and subjective well-being. Path values represent standardized coefficients.

Bias-corrected percentile bootstrapping (5,000 resamples) was used to test the mediating effect of social support (Table 4). The total effect of digital literacy on subjective well-being was 0.085 [95% CI (0.074, 0.096)], the direct effect was 0.062 [95% CI (0.050, 0.074)], and the indirect effect through social support was 0.023 [95% CI (0.017, 0.029)]. The 95% confidence interval for the indirect effect excluded zero (*p* < 0.001), indicating that social support partially mediated the relationship between digital literacy and subjective well-being, with the mediation effect accounting for 27.1% of the total effect Figure 2.

**Table 4:** Mediation effect analysis (process model 4).

Path	Symbol	Effect	95% CI	SE	*P*
Digital literacy → social support → subjective well-being	a × b	0.023	0.017 ~ 0.029	0.003	**<0.001**
Digital literacy → social support	a	0.207	0.182 ~ 0.231	0.012	**<0.001**
Social support → subjective well-being	b	0.110	0.086 ~ 0.134	0.012	**<0.001**
Digital literacy → subjective well-being (direct)	c’	0.062	0.050 ~ 0.074	0.006	**<0.001**
Digital literacy → subjective well-being (total)	c	0.085	0.074 ~ 0.096	0.005	**<0.001**

*p*<0.001 suggests all aforementioned causal paths have strong statistical reliability. The study results are robust and not vulnerable to random sampling variation.

## 4 Discussion

### 4.1 Analysis of digital literacy, subjective well-being, and social support among older adults

The digital literacy score of older adults in this study was 52.58 ± 13.19, indicating a moderately low overall level. Demographic analysis revealed that higher educational attainment was associated with significantly higher digital literacy scores, a finding consistent with Jung et al. (22) cross-sectional study of 869 Korean older adults (aged ≥65 years), which identified education as a core predictor of digital literacy across digital access, digital competency, and digital utilization dimensions. Cross-national comparisons from Germany, Japan, and Thailand further confirmed education as a key structural factor influencing the digital divide, with lower-educated older adults facing more pronounced barriers in digital skills acquisition (23).

Regarding age, participants aged 60–69 years scored significantly higher than those aged 70–79 years, reflecting the objective reality that “the older the age, the more difficult the digital integration.” Niu attributed this phenomenon to “digital anxiety”—as age increases, older adults’ fear of technical errors, concerns about information overload, and worries about privacy and security intensify, creating psychological barriers that impede digital integration (24). This observation aligns with findings from Germany and Japan, where internet usage rates among adults aged 75 years and older were significantly lower than among younger older adults, with age itself becoming a more important predictor than income or region (25).

The subjective well-being score (51.84 ± 6.26) and social support score (44.34 ± 3.38) were relatively low, suggesting that despite accelerated digitalization, older adults’ social support networks remain weak. Indeed, social isolation and loneliness have emerged as public health challenges in the context of global aging. Tohyama et al. systematic review explicitly identified social frailty—including lack of social support, reduced social participation, and deficiency of social resources—as an important predictor of health outcomes in older adults, with digital interventions regarded as a potential strategy to reverse social vulnerability (26).

### 4.2 Positive effect of digital literacy on subjective well-being

Correlation analysis demonstrated a strong positive association between digital literacy and subjective well-being (*r* = 0.943, *p* < 0.01). Regression analysis further confirmed that, after controlling for demographic variables, digital literacy significantly predicted subjective well-being (*β* = 0.401, *p* < 0.01). Jung et al. found that digital literacy positively influenced life satisfaction among Korean older adults, with the effect remaining robust after demographic adjustment. This study particularly emphasized that the effect of digital literacy extends beyond operational skills to digital competency and digital utilization dimensions—that is, whether older adults can effectively integrate digital tools into daily life to gain tangible benefits in convenience and social participation (22). Niu further extended this theoretical perspective, arguing that digital literacy in later life functions not merely as a technical skill but as an important form of psychological and social capital, enhancing autonomy, well-being, and social integration among older adults. This study, based on an integrated technology acceptance model and social cognitive theory, confirmed a significant negative association between digital anxiety and usage intention, while achievement experience effectively buffered this negative relationship (24).

From a social support theory perspective, the facilitating effect of digital literacy on subjective well-being can be explained through enhanced social connectedness. Li et al. demonstrated the positive pathways through which digital literacy influences health and quality of life among rural older adults (mediated by social capital) (27). Liang et al.’s systematic review and meta-analysis further provided evidence supporting this perspective (28).

Furthermore, international evidence has increasingly supported the beneficial role of digital engagement for older adults’ well-being. Htoo Pan et al. (29) examined digital inclusion among community-dwelling older adults in Singapore and found that digital skills were significantly associated with health outcomes, emphasizing the need for targeted digital literacy interventions. Augner (30) reported that self-rated computer skills among older adults were positively associated with higher education, better cognitive abilities, and increased mental health, highlighting the cognitive and psychological benefits of digital competence. Mubarak and Suomi (31) drew attention to the “grey digital divide,” noting that elderly individuals are increasingly marginalized in the information age, and called for comprehensive strategies to address digital exclusion among aging populations. These international findings collectively underscore the global relevance of promoting digital literacy as a public health strategy for active aging.

Furthermore, cross-cultural research offers a broader theoretical lens for understanding the relationship between digital literacy and well-being. Comparative studies across Germany, Japan, and Thailand revealed that strategies to promote digital inclusion must be adapted to each country’s cultural context: Germany’s community center model emphasizes individual autonomous learning, Japan’s volunteer network emphasizes collective mutual assistance, and Thailand’s government-led model relies on top-down policy promotion (23). These differences suggest that the effect of digital literacy on well-being is not only contingent on individual capacity but also significantly moderated by sociocultural environments and policy ecosystems.

### 4.3 Mediating mechanism of social support

Social support partially mediated the relationship between digital literacy and subjective well-being, with the mediation effect accounting for 27.1% of the total effect. Specifically, digital literacy enhances subjective well-being by strengthening older adults’ capacity to access social support.

The mediation effect was tested using the PROCESS macro (Model 4) with bias-corrected bootstrapping, which is widely recognized as a robust method for testing mediation hypotheses (21). This approach offers several advantages over traditional causal steps methods: it does not require the assumption of normality for the sampling distribution of the indirect effect, it provides a direct test of the indirect effect through confidence intervals, and it allows for the inclusion of covariates to control for confounding variables. The results demonstrated that the indirect effect of digital literacy on subjective well-being through social support was statistically significant [a × b = 0.023, 95% CI (0.017, 0.029)], confirming the partial mediating role of social support.

From the specific pathways of the mediation mechanism, improved digital literacy may enhance social support through the following avenues: First, digital skills enable older adults to maintain more frequent contact with children, relatives, and friends via social media, obtaining emotional and intergenerational support. Niu found that family support significantly promoted older adults’ intention to use digital tools, with this effect realized through reduced digital anxiety. The study emphasized that in modern family structures where adult children and elderly parents often live separately, digital technology serves as a critical bridge for bridging intergenerational distance and sustaining emotional bonds (18). Second, digital literacy enables older adults to participate in online community activities and interest groups, expanding social networks. Based on data from the 2020 China Longitudinal Aging Social Survey (CLASS), Luo et al. (32) from the perspective of social capital theory, noted that moderate social capital facilitates older adults’ digital integration by expanding social networks and reinforcing norms of reciprocity, with psychological identity playing a key mediating role. Notably, their study revealed an “inverted U-shaped” relationship—excessive social resources may paradoxically challenge older adults’ sense of autonomy and competence, offering a new perspective on the “dose effect” of social support.

Importantly, the acquisition of social support is not entirely passive but is closely associated with older adults’ own health initiative. Using CGSS 2021 data, Tang et al. (33) confirmed that digital literacy influences health behaviors among middle-aged and older adults through the sequential mediation of proactive health awareness and social capital, forming a pathway of “digital literacy → proactive health awareness → social capital → health behavior” (sequential indirect effect = 0.011, *p* < 0.01). Notably, this sequential mediation effect was significant only in the middle-aged group (aged 45–59 years) and not in those aged 60 years and older, suggesting a critical window for digital literacy empowerment and indicating that interventions initiated in midlife may yield better outcomes than delayed interventions in later life.

### 4.4 Theoretical contributions and practical implications

The theoretical contributions of this study are threefold: First, it empirically validates the mediating role of social support between digital literacy and subjective well-being among older adults, conceptualizing digital literacy as a form of psychological and social capital and extending the analytical boundaries of traditional social support theory. Second, it provides empirical evidence from the Chinese context, contributing to the cross-cultural understanding of digital inclusion and active aging. Third, it identifies the partial mediation mechanism, clarifying the direct and indirect pathways through which digital literacy influences well-being.

Based on these findings, the following practical recommendations are proposed: First, digital literacy enhancement for older adults should be incorporated into the core agenda of active aging strategies, with stratified digital skills education delivered through community training programs and senior universities, with particular attention to the oldest-old and lower-educated subgroups. Second, the role of family in digital back-feeding (intergenerational support in technology use) should be fully leveraged. Third, community-based digital literacy training programs have demonstrated promising outcomes in China. For example, the “See Young” youth volunteer initiative, founded by Beijing University of Posts and Telecommunications in 2011, has conducted over 15,000 free training sessions across more than 500 residential communities, reaching millions of older adults. This program emphasizes teaching practical smartphone skills (e.g., photography, instant messaging, and online shopping) tailored to everyday needs, with university student volunteers providing patient, individualized instruction. Similarly, the “Smart Silver” ICT Outreach Programme in Hong Kong employs a multi-pronged approach, including digital device training, VR/AR cognitive activities, and smart health monitoring, to enhance both digital skills and social engagement among elderly participants. These case examples illustrate the feasibility and effectiveness of community-based interventions and provide replicable models for other regions. Fourth, an integrated online-offline social support system for older adults should be established. While advancing digital age-friendly adaptations, offline service channels must be preserved to prevent the “digital divide” from evolving into a “service divide.”

### 4.5 Limitations and future directions

Several limitations must be acknowledged. First, the cross-sectional design precludes causal inference. Second, the mediating variable was assessed only as a total social support score, without differentiating the distinct mediating effects of emotional support, instrumental support, and informational support. Third, although the sample was drawn from six provinces with consideration for regional representativeness, the generalizability of findings requires further empirical testing. Fourth, the digital literacy measurement focused predominantly on skill dimensions, with insufficient examination of psychological variables such as digital anxiety and usage intention. Fifth, the high correlations among the three main variables (*r* > 0.90) may raise concerns about construct overlap. While Harman’s single-factor test and theoretical distinctions between the constructs provide some reassurance, future research should employ longitudinal designs and multi-method assessments (e.g., combining self-report with objective digital skill tests and informant-rated social support) to further validate the discriminant validity of these constructs. Sixth, while the PROCESS macro provides a robust framework for testing mediation, it does not estimate a full structural equation model with latent variables. Future research may benefit from using structural equation modeling with latent variables to further validate the measurement and structural relationships among these constructs. Future research should employ longitudinal designs, incorporate multidimensional measures of social support, and investigate the differential effects of specific digital tools and applications on well-being outcomes.

## 5 Conclusion

This study examined the relationships among digital literacy, social support, and subjective well-being among 1,260 Chinese older adults. The findings indicate that digital literacy among older adults is at a moderately low level (52.58 ± 13.19) and is significantly influenced by educational attainment and age. Digital literacy was found to positively predict subjective well-being (*β* = 0.401, *p* < 0.001), supporting Hypothesis 1. Digital literacy also positively predicted social support (*β* = 0.428, *p* < 0.001), supporting Hypothesis 2. Most importantly, social support was found to partially mediate the relationship between digital literacy and subjective well-being, with the indirect effect accounting for 27.1% of the total effect [indirect effect = 0.023, 95% CI (0.017, 0.029)], supporting Hypothesis.

The findings carry important implications for active aging strategies. Enhancing digital literacy among older adults represents a viable pathway to promote subjective well-being, both directly and indirectly through strengthened social support networks. Policymakers and practitioners should prioritize community-based digital literacy programs, leverage intergenerational digital support, and establish integrated online-offline social support systems to ensure that the benefits of digitalization are equitably distributed across the aging population. Future longitudinal and intervention studies are warranted to establish causal relationships and to identify the most effective components of digital literacy training for older adults.

## Data Availability

The original contributions presented in the study are included in the article/supplementary material, further inquiries can be directed to the corresponding author.
